# Inversin/Nephrocystin-2 Is Required for Fibroblast Polarity and Directional Cell Migration

**DOI:** 10.1371/journal.pone.0060193

**Published:** 2013-04-08

**Authors:** Iben R. Veland, Rodrick Montjean, Lorraine Eley, Lotte B. Pedersen, Albrecht Schwab, Judith Goodship, Karsten Kristiansen, Stine F. Pedersen, Sophie Saunier, Søren T. Christensen

**Affiliations:** 1 Department of Biology, University of Copenhagen, Copenhagen, Denmark; 2 Inserm U-983, Imagine Institut, Paris Descartes-Sorbonne Paris Cité University, Necker Hospital, Paris, France; 3 Institute of Human Genetics, Newcastle University, Newcastle upon Tyne, United Kingdom; 4 Institute of Physiology II, Münster University, Münster, Germany; National Cancer Center, Japan

## Abstract

Inversin is a ciliary protein that critically regulates developmental processes and tissue homeostasis in vertebrates, partly through the degradation of Dishevelled (Dvl) proteins to coordinate Wnt signaling in planar cell polarity (PCP). Here, we investigated the role of Inversin in coordinating cell migration, which highly depends on polarity processes at the single-cell level, including the spatial and temporal organization of the cytoskeleton as well as expression and cellular localization of proteins in leading edge formation of migrating cells. Using cultures of mouse embryonic fibroblasts (MEFs) derived from *inv^−/−^* and *inv^+/+^* animals, we confirmed that both *inv^−/−^* and *inv^+/+^* MEFs form primary cilia, and that Inversin localizes to the primary cilium in *inv^+/+^* MEFs. In wound healing assays, *inv^−/−^* MEFs were severely compromised in their migratory ability and exhibited cytoskeletal rearrangements, including distorted lamellipodia formation and cilia orientation. Transcriptome analysis revealed dysregulation of Wnt signaling and of pathways regulating actin organization and focal adhesions in *inv^−/−^* MEFs as compared to *inv^+/+^* MEFs. Further, Dvl-1 and Dvl-3 localized to MEF primary cilia, and β-catenin/Wnt signaling was elevated in *inv^−/−^* MEFs, which moreover showed reduced ciliary localization of Dvl-3. Finally, *inv^−/−^* MEFs displayed dramatically altered activity and localization of RhoA, Rac1, and Cdc42 GTPases, and aberrant expression and targeting of the Na^+^/H^+^ exchanger NHE1 and ezrin/radixin/moesin (ERM) proteins to the edge of cells facing the wound. Phosphorylation of β-catenin at the ciliary base and formation of well-defined lamellipodia with localization and activation of ERM to the leading edge of migrating cells were restored in *inv^−/−^* MEFs expressing Inv-GFP. Collectively, our findings point to the significance of Inversin in controlling cell migration processes, at least in part through transcriptional regulation of genes involved in Wnt signaling and pathways that control cytoskeletal organization and ion transport.

## Introduction

Inversin (Inv or Nephrocystin-2) is encoded by the *inversion of embryo turning* (*invs)* gene [1]–[3] and was first discovered for its role during mammalian embryonic development in establishment of left-right asymmetry [4], which is reversed (*situs inversus*) in *inv^−/−^* mice with a homozygous deletion of exons 4–12, rendering only the first three exons transcribed [5]. Apart from laterality defects, the *inv^−/−^* mice exhibit cardiac, liver and kidney anomalies, including cyst formation in the extrahepatic bile ducts, pancreas and kidneys [1], [3], [4], [6]–[9]. In humans, *INVS* was identified as the gene encoding Nephrocystin-2 (Nphp2) that is mutated in the recessive cystic kidney disease nephronophthisis type 2/infantile nephronophthisis [2], which, to a variable extent, is accompanied by *situs inversus* and other phenotypic traits of the *inv^−/−^* mouse and retinitis pigmentosa [10], [11].

The four known mammalian Inversin splice variants of 90, 125, 140 and 165 kDa localize in a cell cycle-dependent manner to cell edges and primary cilia during growth arrest [12]–[14]. Primary cilia are microtubule-based, sensory organelles that emanate from the centrosomal mother centriole and coordinate a series of signaling pathways such as Hedgehog, Wnt and receptor tyrosine kinase (RTK) signaling during embryonic development and in tissue homeostasis [15]–[17]. Consequently, defects in the formation or function of primary cilia lead to a series of genetic disorders and diseases now commonly known as ciliopathies, including laterality defects, congenital heart disease, cystic kidney diseases and retinitis pigmentosa [18], [19]. Endogenous Inversin was reported to localize to a confined region in the proximal segment of the primary cilium of mouse epithelial cells, referred to as the inv compartment [20]. Here, Inversin interacts with other Nphp proteins to form complexes with Meckel-Gruber syndrome (MKS) and Joubert syndrome (JBS) proteins that control trafficking and signaling properties of the primary cilium [21]–[24].

Wnt signaling has been associated with the primary cilium due to the localization of several Wnt signaling components, including Inversin, at the ciliary/centrosomal axis in both hESC and differentiated cells [25]–[29]. Wnt signaling is initiated by the binding of a Wnt ligand to a Frizzled (Fzd) receptor and co-receptors, and has traditionally been divided into canonical and non-canonical Wnt pathways. Canonical Wnt signals rescues β-catenin from degradation by a complex comprising Glycogen Synthase Kinase 3 beta (GSK3β), Axin, Casein Kinase 1 (CK1) and adenomatous polyposis coli (APC), in turn leading to β-catenin-mediated gene transcription. In contrast, non-canonical Wnt signaling operates independently of β-catenin to control planar cell polarity (PCP) that refers to the organization of cells within the plane of a tissue [30]–[33].

PCP appears to be a prerequisite for correct cilia formation, yet Inversin may play a critical role in regulating Wnt signaling at the primary cilium to control PCP (discussed in [33], [34]). In *inv^−/−^* mice, the hair patterning phenotype is reminiscent to that of PCP defects, and convergence extension movements are impaired by knockdown of Inversin in *Xenopus laevis* embryos [35]. Inversin was suggested to promote non-canonical Wnt signaling by recruiting or stabilizing Dishevelled (Dvl) at the plasma membrane in response to Fzd8 signal during *Xenopus* pronephros development [36]. Furthermore, Inversin was found to inhibit canonical signaling by degradation of Dvl-1 via the proteasome [35], which is hypothesized to localize at the ciliary base [37]. In support of these findings, elevated responsiveness to Wnt signals was reported in vertebrate cells with absent or incomplete primary cilia [24], [27], [31].

Mounting evidence links Wnt signaling, and in particular Dvl proteins, to control of the activity of Rho GTPases RhoA and Rac [38], [39], but a role for Inversin in regulation of Rho GTPases has so far not been directly addressed. Cell motility and cytoskeletal organization are strongly influenced by the Rho GTPases [40]–[42], with Cdc42 and Rac1 involved predominantly in the formation of filopodia and lamellipodia, and RhoA in stress fiber formation [43]. In addition, an important reciprocal regulatory interaction exists between RhoA and the ezrin/radixin/moesin (ERM) family proteins, which play essential roles in single-cell polarization and cell migration [44]; yet, the possible link between Inversin and ERM proteins is unknown. Further, it is uncertain how Inversin regulates the reorganization of organelles and cytoskeleton during polarity processes and cell migration, and whether this relates to the function of Inversin in the primary cilium. Interestingly, primary cilia were found to orient in parallel to the migratory direction and towards the leading edge of fibroblasts and smooth muscle cells [45]–[47], suggesting that orientation of the cilium is part of the polarity system that transmits positional cues to migrating cells [48]. We recently showed that the primary cilium is required for directional cell migration in fibroblasts, in part by coordinating PDGFRαα-signaling [45] that regulates the spatial organization of translocation, incorporation and activation of the Na^+^/H^+^ exchanger 1 (NHE1) at the leading edge of the migrating cells [49], [50]. NHE1 interacts directly with ERM proteins, is regulated by Rho GTPases, and in turn regulates leading edge Cdc42 activity, cytoskeletal organization, cell adhesion and migration [51]. Hence, the function and localization of NHE1 are potentially linked to Inversin signaling.

Here, we investigated the involvement of Inversin in control of directional cell migration by comparing wt mouse embryonic fibroblasts (MEFs) with MEFs derived from the *inv^−/−^* mouse. In wound healing assays, *inv^−/−^* MEFs exhibit reduced lamellipodia formation and cell migration compared to *inv^+/+^* MEFs, in conjunction with disorientation of the primary cilium. We also show that Inversin, Dvl-1 and Dvl-3 localize to MEF primary cilia, and that canonical Wnt signaling is elevated in *inv^−/−^* MEFs, which furthermore show reduced ciliary localization of Dvl-3. This is associated with differential expression of genes involved in Wnt signaling and cytoskeletal organization, and altered regulation of RhoA, Rac1, Cdc42, NHE1, and ERM proteins. Collectively, our findings point to the significance of Inversin in controlling the migratory behavior of fibroblasts, at least in part through transcriptional regulation of genes involved in Wnt/PCP signaling and pathways that control cytoskeletal organization and ion transport.

## Materials and Methods

### Isolation of *inv^+/+^* and *inv^−/−^* MEFs

Mouse embryonic fibroblasts (MEFs) were established from *inv^−/−^* and *inv^+/+^* E13.5 embryos. Animals were held according to Home office guidelines to maintain health and ensure welfare. Animals were housed in IVC cages and supplied with food and water ad libitum and provided with environmental enrichment to enhance their standard of living and avoid stereotypical behaviors (such enrichment included sunflower seeds - encourages foraging behavior, torn paper bedding - encourages nest building and feeling of security, mouse houses - again encourages security and wellbeing, bacon pellets - foraging and also variation in diet). Methods of sacrifice were as per the Home Office Schedule 1 regulations and are detailed as follows; pregnant females were exposed to carbon dioxide in a rising concentration and death ensured by neck dislocation. The embryos were isolated and placed in ice cold PBS and subsequent decapitation carried out. Newcastle Ethics Review Committee approved this study.

Viscera, liver and heart were discarded from the embryos, and the remaining embryo was cut into fine pieces in the presence of Trypsin-EDTA (Invitrogen). Further Trypsin-EDTA was added and the digested tissue was incubated in a Petri dish at 37°C, 95% humidity and 5% CO_2_ until individual cells were visible under microscope. In a 50 ml tube, the digested tissue was mixed with 37°C Dulbecco's Modified Eagle’s Medium (DMEM, high glucose without sodium pyruvate), 10% heat-inactivated foetal bovine serum (FBS), non essential amino acids, and 50 U/ml penicillin/streptomycin (all from Gibco)) and allowed to settle. The top layer was separated from the bottom layer and cultured in a T25 tissue culture flask (Cellstar) for future experiments. The bottom layer was placed in a separate tissue flask and kept as a backup.

### Cell Culture

*inv^+/+^* and *inv^−/−^* MEFs were grown in 45% DMEM, 45% F-12+L-Glutamine with 10% FBS and 50 U/ml penicillin/streptomycin (all from Gibco) in T75 flasks (Cellstar) at 37°C, 95% humidity and 5% CO_2_. The cells were passaged every 3-4 days by trypsination (Trypsin-EDTA, Gibco) at a confluence of ∼80% and were never used beyond passage 15 to minimize clonal selection. Growth arrest and cilia formation in experimental cells were induced as described previously [52] by growing cells to 90% confluence and replacing the growth medium with a serum-free equivalent for 24–48 h after 2×wash in PBS.

### Characterization of *inv^+/+^* and *inv^−/−^* MEFs by qPCR Analysis

RNA from *inv^+/+^* versus *inv^−/−^* MEFs was purified with a Qiagen Extraction Kit, treated with DNase I, and one µg of total RNA was reverse-transcribed using Superscript II (Invitrogen). Relative expression levels of the *Nphp2* mRNA were determined by quantitative RT–PCR using Absolute SYBR Green ROX Mix (*ABgene*) and a set of primers specific for the *Nphp2* gene (forward primer: 5′-ACTTGTTACCCAGCATATGTGGTC-3′, reverse primer: 5′-AGGAGAAAACATTTGAACCTTGTCTT-3′). *Nphp2* expression was normalized to *Gapdh* mRNA expression (forward primer: 5′-TGCACCACCAACTGCTTAG-3′, reverse primer: 5′- GGATGCAGGGATGATGTTC-3′). Data were analyzed with the 2^−ΔΔCt^ method and values are expressed as the average of triplicates. Levels of *Nphp2* expression in the different MEFs were normalized to those of wt MEF cells and confirmed the essential absence of Inversin in *inv^−/−^* MEFs (Figure S1).

### Transcriptome Analysis

Cycling or growth arrested cells were lysed in lysis buffer with 1% β-mercaptoethanol, and RNA was purified using the NucleoSpin RNA II kit (Macherey-Nagel, cat. no. 740955-50), as recommended by the manufacturer. Digital Gene Expression tag profiling (DGE) was performed essentially as previously described [53] using Illumina’s Digital Gene Expression Tag Profiling Kit according to the manufacturer’s protocol (version 2.1B). One µg of RNA was used for preparation of cDNA on magnetic Oligo(dT) beads using Superscript II (Invitrogen). The cDNA was digested with *Nla*III, ligated to GEX *Nla*III Adapter 1, digested with *Mme*I, ligated to GEX Adapter 2 and amplified by PCR. Amplicons were gel purified. Cluster formation was performed according to Illumina’s instruction using a DNA concentration of 1–4 pM and amplification for 35 cycles. Sequencing was conducted on the Illumina Genome Analyzer II (GAII). Tags were filtered and alignment against the mouse genome was performed using SOAP [54] and normalized to 1M tags. Functional annotation was performed using DAVID Tools (http://david.abcc.ncifcrf.gov/summary.jsp). Gene Set Enrichment Analysis (GSEA) was performed using Broad Institute software (http://www.broadinstitute.org/gsea/index.jsp).

### Transfections

Cells were transfected with a BosEx vector containing full length mouse Inversin fused to green fluorescent protein (Inv-GFP) [55] kindly provided by Dr. Hiroshi Hamada, Osaka University, Japan. Transfections were carried out as previously described, either by nucleofection with the Nucleofector device II from Amaxa Biosystems [56] or using DharmaFECT transfection reagent (Thermo Scientific) [57]. For nucleofection, 2×10^6^ suspended cells were nucleofected with 2 µg plasmid DNA in MEF2 buffer. For DharmaFECT transfections, cells were grown to 50% confluence on coverslips in six well trays and incubated four hours with 1.5 µg plasmid DNA premixed with 5 µl DharmaFECT under serum free conditions. Subsequently, the cells were cultured and treated as described below.

### Antibodies and Staining Reagents

Primary antibodies: mouse anti-acetylated α-tubulin, Sigma (T7451); mouse anti-β-actin, Sigma (A5441); mouse anti-Adenomatous poliposis coli, Santa Cruz (SC-53165); rabbit anti-β-catenin, Epitomics (1247-S); rabbit anti-phospho-β-catenin, Cell Signaling (9561S); rabbit anti-Cdc42, Cell Signaling (2462S); goat anti-centrin2, Santa Cruz (SC-8025); rabbit anti-detyrosinated α-tubulin (Glu-tubulin), AbCam (AB48389); mouse anti-Dishevelled-1, Santa Cruz (SC-8025); rabbit anti-Dishevelled-2, Cell Signaling (3224); rabbit anti-Dishevelled-3, Cell Signaling (3218); mouse anti-ezrin, Cell Signaling (E8897); rabbit anti-ezrin/radixin/moesin, Cell Signaling (3142); rabbit anti-phospho-ezrin/radixin/moesin, Cell Signaling (3142); chicken anti-GFP, AbCam (ab13970); mouse anti-GFP, AbCam (ab1218); rabbit anti-GFP, Santa Cruz (sc-8334); mouse anti-GSK3β, BD Transduction, (610201); goat anti-Inversin, Santa Cruz (SC-8719); rabbit anti-moesin, Epitomics (2036-1); mouse anti-NHE1, Santa Cruz (sc-136239); rabbit anti-radixin, Epitomics (2042-1); rabbit anti-phospho-Retinoblastoma protein, Cell Signaling (9308S); mouse anti-vinculin, Sigma (V9131); mouse anti-Rac1, Transduction Laboratories (610651); mouse anti-RhoA, Cytoskeleton (ARH03). The rabbit anti-NHE1 antibody was a kind gift from Dr. Josette Noël at University de Montreal, Canada.

Secondary antibodies and staining reagents for immunofluorescence (all from Molecular Probes): Alexa Flour® 350 Donkey IgG, anti-mouse (A10035), Alexa 350 Flour® Donkey IgG, anti-rabbit (A10039); Alexa Fluor® 488 Donkey IgG, anti-goat (A11055); Alexa Fluor® 568 Donkey IgG, anti-mouse (A10037); Alexa Fluor® 488 Donkey IgG, anti-mouse (A21202); Alexa Fluor® 488 Goat IgG, anti-chicken (A11039); Alexa Fluor® 568 Donkey IgG, anti-rabbit (A21206); Alexa Fluor® 488 Donkey IgG, anti-rabbit (A21206); 4′, 6-diamino-2-phenylindole dichloride (D1306); Alexa Fluor® 350 Phalloidin (A22281); Rhodamine-phalloidin (R415). Secondary antibodies for western blot analysis from Sigma: goat anti-mouse F(ab’)2 specific alkaline phosphatase conjugated (A1293); Goat anti-rabbit F(ab’)2 specific alkaline phosphatase conjugated (A3937), Rabbit anti-goat whole molecule alkaline phosphatase conjugated (A4187).

### Immunofluorescence and DIC Microscopy Analysis

Cells were grown on HCl-rinsed coverslips in six-well trays (TTP) and serum deprived to induce cilia formation, then fixed for differential interference contrast (DIC) and immunofluorescence microscopy analysis (IFM) as previously described [52]. Images were obtained with a cooled CCD Optronics camera on a Nikon-Japan, Eclipse E600 epifluorescence microscope or with a cooled CCD Olympus DP72 camera on an Olympus BX63 epifluorescence microscope. Images were digitally processed for overlays with Adobe Photoshop CS4.

### SDS-PAGE and Western Blotting

Cells were grown in Petri dishes, washed in ice-cold PBS and lysed in hot lysis buffer (0.5% SDS, 1 mM Tris-HCl, 1 mM sodium ortho-vanadate and Complete protease inhibitor cocktail (Santa Cruz)). Cell lysates were homogenized by sonication (PowerMED), and SDS-PAGE and Western blotting was performed as previously described [58]. Band intensities were estimated from densitometric values using Un-SCAN-IT *gel* (Silk Software).

### Migration Assays

*inv^+/+^* and *inv^−/−^* MEFs were grown to 100% confluence (monolayer) in T2 flasks (Falcon) followed by serum deprivation to induce cilia formation, and directional cell migration was analyzed by wound healing assays [59]. Micrographs were obtained in 10 min intervals over a time course of 6 h. The circumferences of cells at the wound edge were marked at each time step throughout the entire image stack using Amira 4.1 (TGS, http://amiravis.com). Net translocation, velocity and movement of the cells into the wound (“x-direction” of a virtual coordinate system) were calculated as in [49]. In this experimental setup, translocation into the y-direction (parallel to the wound) was close to zero for *inv^+/+^* as well as *inv^−/−^* MEFs (data not shown) in accordance with [49].

### GTPase Activity Assays

Cells were maintained in culture in the presence of serum until 100% confluence, then serum-starved for 24 h, scratched (40 times per 150 mm petri dish) and allowed to migrate for 30 min or 4 h as indicated. The relative levels of GTP-bound RhoA, Rac1, or Cdc42 were determined by an effector pulldown assay as described by [60]. Lysis of MEFs and pull down assay were done according to the manufacturer’s instructions (Cytoskeleton). In brief, cells were lysed in ice cold buffer and cell lysates were immediately clarified by centrifugation. An aliquot of cell extract was incubated with the GST-PAK fusion protein (Rac1 and Cdc42 assay) or with the GST-rhotekin fusion protein (RhoA assay). The bead pellet was washed and re-suspended in 2×Laemmli sample buffer. Proteins bound to the beads were separated on a 15% SDS polyacrylamide gel, transferred onto a nitrocellulose membrane, and membranes were blotted with RhoA, Rac1 or Cdc42 antibodies. The band density was quantified by Bio1D software (Vilber Lourmat), and the relative densities of pulled down RhoA and Rac1/Cdc42 were normalized to the total RhoA, Rac1 or Cdc42 (input) in the same sample.

### Statistics

Statistical significance of the obtained data was tested with two-tailed Students *t*-test for ciliation frequencies and WB analysis, one-way analysis of variance (ANOVA) for migration assays, and Mann-Whitney U test for GTPase activity assays. Significance levels are indicates with asterisks (*: p<0.05, **: p<0.001, ***: p<0.0001).

## Results

### Directional Cell Migration is Inhibited in *inv^−/−^* MEFs

**Figure 1:**
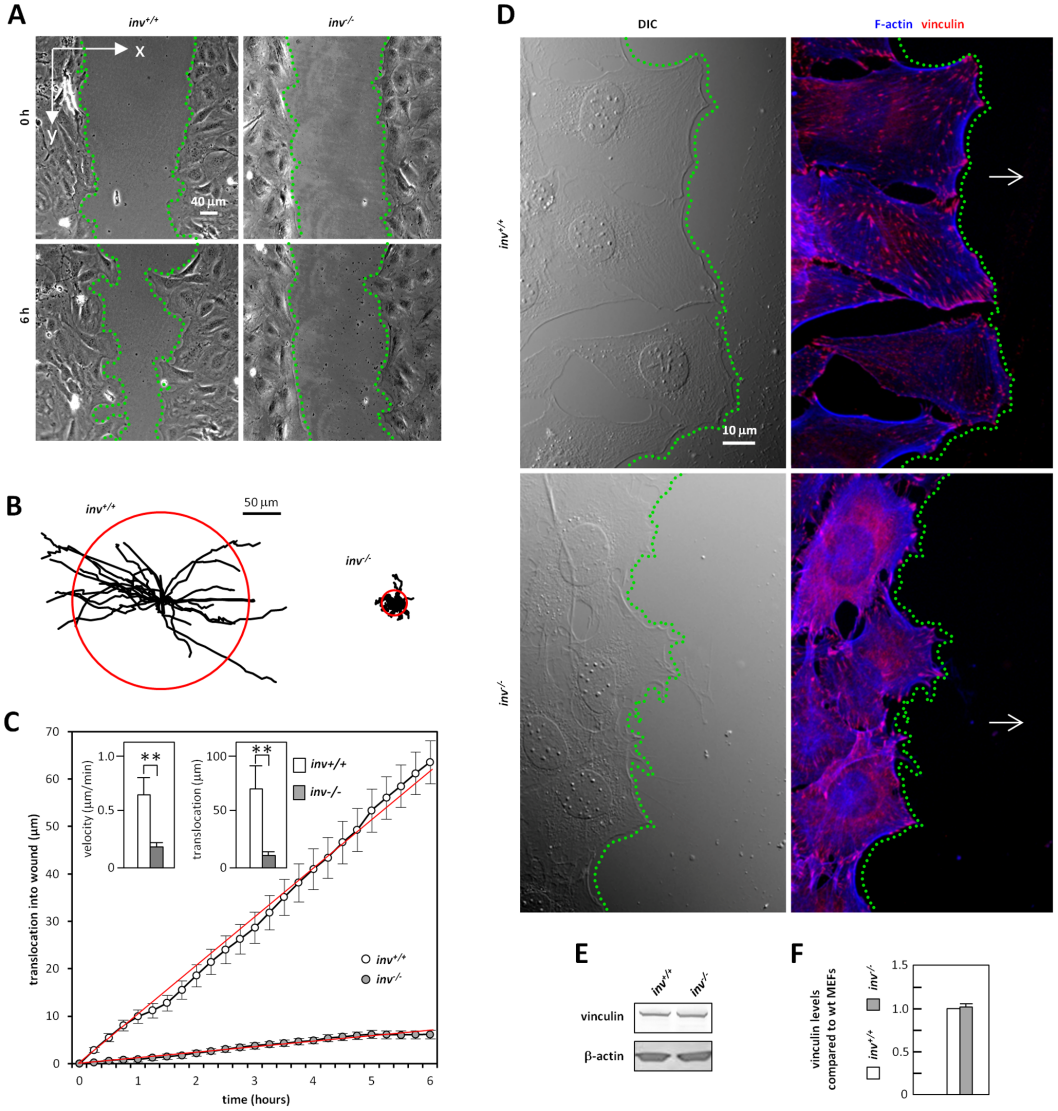
Wound healing assays on cell migration and localization of focal adhesions in growth-arrested *inv^+/+^* and *inv^−/−^* MEFs. Graphs of time-lapse data represent mean values of four independent experiments ± S.E.M. (p<0.001, one-way ANOVA). Cells were serum starved for 24 h and allowed to recover 1 h post wounding, hence t = 0 h in the data sets relates to the initiation of monitoring. (**A**) Light microscopy of *inv^+/+^* and *inv^−/−^* MEFs in wound healing assay at t = 0 h (left) and t = 6 h (right). (**B**) Trajectories of growth-arrested *inv^+/+^* (N = 27) and *inv^−/−^* (N = 28) MEFs in wound healing assay. Each line represents the migration of one cell within a 6 h period. The red circles illustrate the mean translocation of the cells. (**C**) Mean velocity and translocation of cells into the wound. (**D**) IFM analysis of localization of focal adhesions by anti-vinculin (*red*) in cells in wound healing assays and the actin cytoskeleton was stained with phalloidin (F-Actin, *blue*). Open arrows mark direction of migration into the wound and green dotted lines mark the front of cells facing the wound. (**E,F**) SDS-PAGE and WB analysis of expression of vinculin in growth-arrested *inv^+/+^* and *inv^−/−^* MEFs.

The two-dimensional migratory behavior of growth arrested *inv^+/+^* and *inv^−/−^* MEFs was studied using *in vitro* wound healing assays and time-lapse video microscopy. Six h after wound induction, *inv^+/+^* MEFs had migrated more than halfway into the wound, while the *inv^−/−^* MEFs had barely moved (Figure 1A). Figure 1B shows the migratory tracks of *inv^+/+^* and *inv^−/−^* MEFs over 6 h. All trajectories were normalized to a common starting point, with the radius of each red circle representing the average cell displacement after 6 h. As shown in the left hand insert in Figure 1C, the overall velocity of *inv^−/−^* MEFs was about 25% of that in *inv^+/+^* cells, whereas the distance traveled was less than 15% (right hand insert of Figure 1C). The average velocity of directionally migrating cells was obtained as the slope of translocation into the wound (x-direction, see Figure 1A) plotted against time (Figure 1C), and for *inv^−/−^* MEFs, this was approximately 10% of that of the wt (Figure 1C). We next evaluated the ability of the cells to form leading edge lamellipodia, filopodia, and focal adhesions. In contrast to *inv^+/+^* MEFs, *inv^−/−^* MEFs failed to form well-defined lamellipodia whereas filopodia formation appeared largely unaffected. *Inv^−/−^* MEFs also displayed a more diffuse distribution of actin bundles and focal adhesions as detected with anti-vinculin compared to *inv^+/+^* cells (Figure 1D), although total vinculin expression was not altered (Figure 1E,F). Thus, *inv^−/−^* MEFs are severely compromised in their ability to migrate and this is associated with defective formation of leading edge lamellipodia.

### Characterization of Primary Cilia in *inv^+/+^* and *inv^−/−^* MEFs

**Figure 2:**
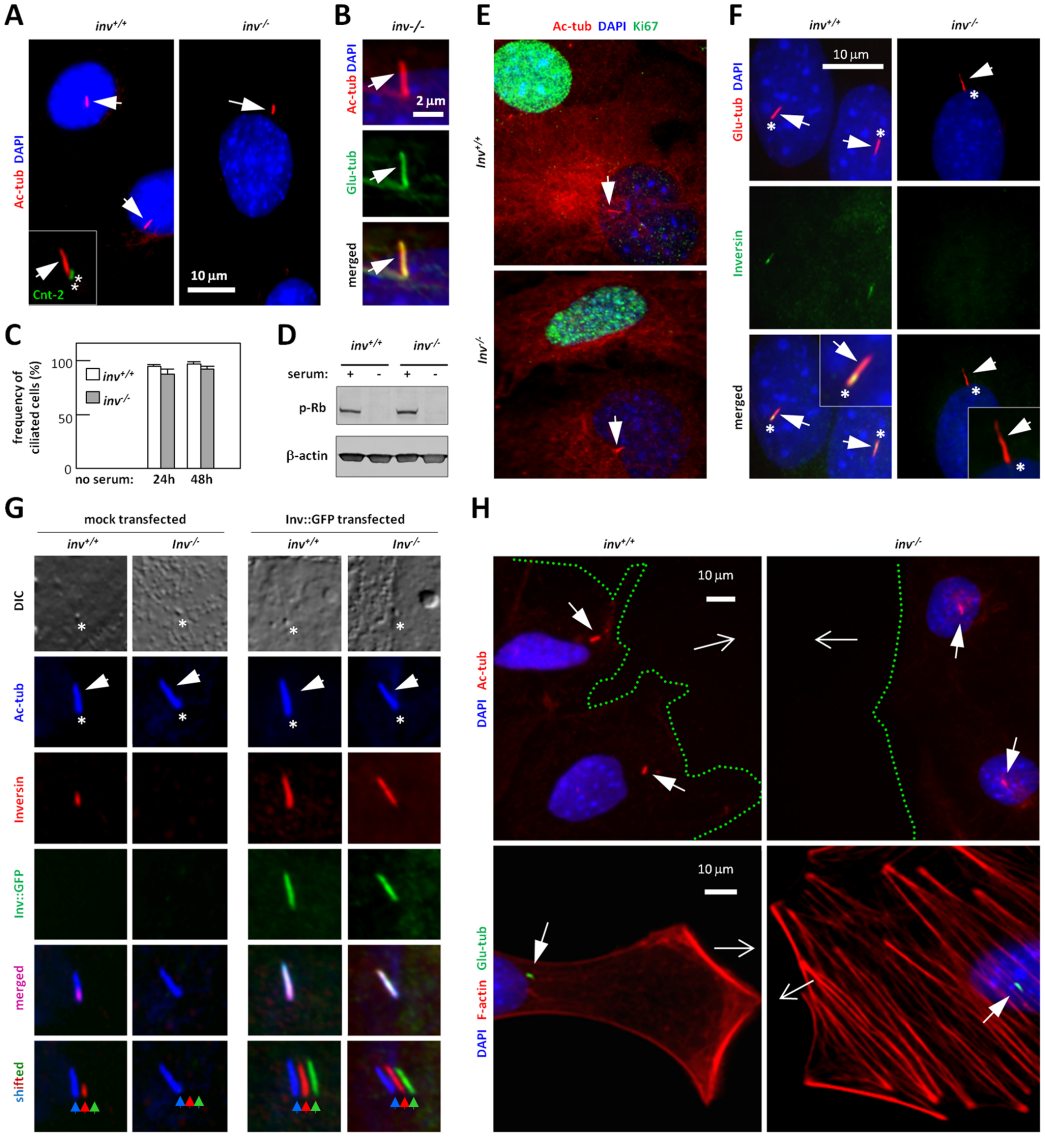
Formation and orientation of primary cilia and localization of Inversin in growth-arrested *inv^+/+^* and *inv^−/−^* MEFs. (**A**) IFM analysis of primary cilia (closed arrows) with anti-acetylated alpha tubulin (Ac-tub, *red*) and their basal body region with anti-Centrin-2 that marks the two centrioles (asterisks) of the centrosome (Ctn-2, *green*). (**B**) IFM analysis of primary cilia (closed arrows) co-labelled with Ac-tub (*red*) and anti-glutamylated alpha-tubulin (Glu-tub, *green*). (**C**) Ciliation frequencies of *inv^+/+^* and *inv^−/−^* MEFs upon 24 and 48 h of serum-free incubation, represented as mean ± S.E.M. (n = 3). (**D**) WB analysis of *inv^+/+^* and *inv^−/−^* MEFs in the presence (+) and absence (−) of serum with anti-phospho-Retinoblastoma protein, which is downregulated in growth arrested cells. **E:** IFM analysis of primary cilia (Ac-tub, *red*, and closed arrows) formation in growth arrested cells. Nuclei (DAPI, *blue*) of cycling cells shows localization of anti-Ki67 (*green*). (**F**) IFM analysis of Inversin (*green*) localization to primary cilia (Glu-tub, *red*, closed arrows) of *inv^+/+^* and *inv^−/−^* MEFs. Asterisks (*) indicate ciliary base, nuclei are stained with DAPI (*blue*). (**G**) IFM analysis of Inv-GFP (*green*) and Inversin (*red*) localization to primary cilia (Ac-tub, *blue*, closed arrows) in mock and Inv::GFP transfected cells. The base of the cilium is identified by Differential interference constrast microscopy, DIC (asterisks). (**H**) IFM analysis of *inv^+/+^* and *inv^−/−^* in wound healing assays after 30 min (top panel) and 4 h (lower panel) migration. Open arrows indicate direction of migration. Primary cilia are stained with Ac-tub (upper panel, *red*, closed arrows) or Glu-Tub (lower panel, *green*) and nuclei are stained with DAPI (*blue*). In lower panel, the actin cytoskeleton is stained with phalloidin (F-actin, *red*). Green dotted lines mark the edge of cells facing the wound.

Immunofluorescence microscopy (IFM) analysis with acetylated α-tubulin (Ac-tub) and detyrosinated α-tubulin (Glu-tub) antibodies showed that both wt and *inv^−/−^* MEFs form primary cilia of approximately 5 µm in length with a well-defined ciliary basal region, which was marked with Centrin antibody (Figure 2A,B). Cilia were formed at a frequency of about 90% after both 24 and 48 h of serum deprivation and in *inv^+/+^* and *inv^−/−^* MEFs (Figure 2C). Both *inv^+/+^* and *inv^−/−^* MEFs entered growth arrest upon serum deprivation as evidenced by a clear reduction in S^807/811^ phosphorylation of Retinoblastoma protein (Rb) (Figure 2D), and ciliary formation occurred only in cells negative for nuclear localization of the proliferation-marker, Ki67 (Figure 1E). These results show that Inversin is not required for cells to enter growth arrest and form primary cilia.

In accordance with previous reports [20], IFM analysis showed localization of endogenous Inversin in wt MEFs to the lower part of the cilium, which signifies the Inversin compartment [20] (Figure 2F). No corresponding immunoreactivity was observed in *inv^−/−^* MEFs, confirming the Inversin specificity of the antibody. As a further control, cells were transfected with Inv-GFP that localized to primary cilia in both *inv^+/+^* and *inv^−/−^* MEFs (Figure 2G). In some cases, Inv::GFP localized to the entire length of the cilium as well as to the ciliary base region (data not shown), which may indicate the dynamic trafficking of the protein to and from the Inversin compartment in cells expressing high levels of the construct. We next evaluated the ability of the primary cilia to reorient in the direction of migration, as previously shown for migrating fibroblasts [45], [46]. In *inv^+/+^* MEFs, the primary cilia were positioned in front of the nucleus and oriented towards the leading edge. In marked contrast, primary cilia of *inv^−/−^* MEFs appeared randomly positioned and lacked an axis of orientation towards the edge of the wound (Figure 2H). These results indicate that Inversin critically regulates the orientation and correct positioning of the cilium, which is part of the polarity process required for cell migration.

### Aberrant Wnt/β-catenin Signaling in *inv^−/−^* MEFs

In order to delineate the mechanisms through which Inversin exerts its effects on directional cell migration, we initially performed transcriptome analysis on total RNA isolated from quiescent *inv^+/+^* and *inv^−/−^* MEFs. As listed in Table 1, the analysis showed a significant change in the expression of Wnt signaling related genes in *inv^−/−^* MEFs, as evidenced by a major change in the expression profile of genes encoding Wnt ligands, ligand scavengers and secreted proteins that impact on Wnt signaling (*Rspo1*, *Sfrp1, Srfp2, Dkk3*), Wnt receptors and co-receptors (*Fzd2*, *Fzd5*, *Ror2*), various isoforms of casein kinases (*Csnk2a1*, *Csnk2b*, *Csnk1g2*), cyclins (*Ccnd1, Ccnd2*), and protein phosphatases (*Ppp2r1a*, *Ppp2r5d*, *Ppp3r1*, *Ppp4r1*), as well as transcriptional regulators (*Chd8*, *Gsc*, *Fosl1*, *Tcf4*, *Tcf7l1*). Interestingly, we found an upregulation of several β-catenin target genes, such as *Sfrp2* [61], *Tcf4* [62], *Tcf7l1*, *Wisp2* [63], *Ccnd1* [64], [65], *Ccnd2* [66] and *Gsc* [67] in *inv^−/−^* MEFs, consistent with increased canonical Wnt/β-catenin signaling in these cells. These findings are in agreement with previous studies showing that Inversin acts as a molecular switch between Wnt signaling pathways, by inhibiting Wnt/β-catenin signaling via degradation of cytoplasmic Dvl-1 [35]. The transcriptome analysis further revealed a major change in the expression profile of those Wnt signaling-related genes that regulate pathways associated with actin cytoskeleton reorganization and targeting of receptors and regulatory proteins to the leading edge of migrating cells. For instance, *Nherf1*, encoding Na^+^/H^+^ exchange regulatory factor-1, which inhibits β-catenin activity through the tethering of Fzd to the actin cytoskeleton [68] and functions as an adaptor protein for ezrin/radixin/moesin (ERM) proteins and activator of Na^+^/H^+^ exchanger 1 (NHE1) to control directional cell migration [69]–[71], is strongly downregulated in *inv^−/−^* MEFs.

**Table 1:** Transcriptomic analysis of up- and down-regulated genes in growth arrested *inv^−/−^* relative to *inv^+/+^* MEFs with listed *p* values (n = 3) and common protein names.

Gene	Log2 ratio(*inv^−/−/^inv^+/+^*)	*p*-value	Transcript ID	Protein name
***Rspo1***	−8.37	(0.000)	gi|227452350|ref|NM_138683.2|	**R-spondin 1**
***Grem1***	−7.11	(0.000)	gi|215490119|ref|NM_011824.4|	**Gremlin 1**
***Ror2***	−5.19	(0.000)	gi|47271532|ref|NM_013846.3|	**Receptor tyrosine kinase-like orphan receptor 2**
***Nherf1***	−4.05	(0.000)	gi|115270973|ref|NM_012030.2|	**Na^+^/H^+^ exchange regulatory factor 1**
***Tm4sf1***	−3.65	(0.000)	gi|88900519|ref|NM_008536.3|	**Transmembrane 4 L six family member 1**
***Sfrp1***	−2.93	(0.000)	gi|227908833|ref|NM_013834.3|	**Secreted frizzled-related protein 1**
***Csnk2a1***	−1.87	(0.000)	gi|164565453|ref|NM_007788.3|	**Casein kinase 2, alpha 1 subunit**
***Daam2***	−1.54	(0.000)	gi|116089317|ref|NM_001008231.2|	**Dishevelled associated activator of morphogenesis 2**
***Apc2***	−1.46	(0.000)	gi|117938321|ref|NM_011789.2|	**Adenomatosis polyposis coli 2**
***Ppp2r5d***	−1.43	(0.000)	gi|226958535|ref|NM_009358.3|	**Protein phosphatase 2, regulatory subunit B, delta**
***Fbxw4***	−1.43	(0.000)	gi|118130948|ref|NM_013907.2|	**F-box/WD repeat-containing protein 4**
***Cdk14***	−1.41	(0.000)	gi|161086910|ref|NM_011074.2|	**Cyclin-dependent kinase 14**
***Chd8***	−1.34	(0.000)	gi|119392063|ref|NM_201637.2|	**Chromodomain helicase DNA binding protein 8**
***Fzd5***	−1.34	(0.000)	gi|111160872|ref|NM_001042659.1|	**Frizzled 2**
***Csnk2b***	−1.18	(0.000)	gi|118129820|ref|NM_009975.2|	**Casein kinase 2, beta subunit**
***Ppp2r1a***	−1.18	(0.000)	gi|118131166|ref|NM_016891.3|	**Protein phosphatase 2, regulatory subunit A, alpha**
***Ppp3r1***	−1.18	(0.000)	gi|84794596|ref|NM_024459.2|	**Protein phosphatase 3, regulatory subunit B, alpha**
***Fosl1***	−1.12	(0.000)	gi|118129965|ref|NM_010235.2|	**Fos-related antigen 1**
***Nbl1***	−1.10	(0.000)	gi|148234600|ref|NM_008675.2|	**Neuroblastoma, suppression of tumorigenicity 1**
***Ppp4r1***	−1.07	(0.000)	gi|166706859|ref|NM_146081.2|	**Protein phosphatase 4, regulatory subunit 1**
				
***Gsc***	10.61	(0.000)	gi|6754075|ref|NM_010351.1|	**Goosecoid**
***Sfrp2***	5.47	(0.000)	gi|214010193|ref|NM_009144.2|	**Secreted frizzled-related protein 2**
***Dkk3***	5.01	(0.000)	gi|31560475|ref|NM_015814.2|	**Dickkopf-related protein 3**
***Tspan12***	4.41	(0.003)	gi|146198700|ref|NM_173007.3|	**Tetraspanin 12**
***Prickle2***	4.10	(0.000)	gi|197333828|ref|NM_001081146.2|	**Prickle homolog 2**
***Rspo3***	2.88	(0.000)	gi|269973855|ref|NM_028351.3|	**R-spondin 3**
***Tcf4***	2.27	(0.000)	gi|145386521|ref|NM_001083967.1|	**Transcription factor 4**
***Wisp2***	1.73	(0.000)	gi|255683442|ref|NM_016873.2|	**Wnt-1-inducible signaling parthway protein 2**
***Ccnd2***	1.70	(0.000)	gi|80751174|ref|NM_009829.3|	**Cyclin D2**
***Ccnd1***	1.51	(0.000)	gi|119672895|ref|NM_007631.2|	**Cyclin D1**
***Pygo2***	1.50	(0.000)	gi|142351456|ref|NM_026869.2|	**Pygopus homolog 2**
***Cul1***	1.50	(0.000)	gi|34328459|ref|NM_012042.3|	**Cullin 1**
***Csnk1g2***	1.48	(0.000)	gi|227430312|ref|NM_001159591.1|	**Casein kinase 1, gamma 2 subunit**
***Tcf7l1***	1.41	(0.000)	gi|87044902|ref|NM_009332.2|	**Transcription factor 7-like 1**
***Fzd2***	1.01	(0.000)	gi|125628663|ref|NM_020510.2|	**Frizzled 5**

**Figure 3:**
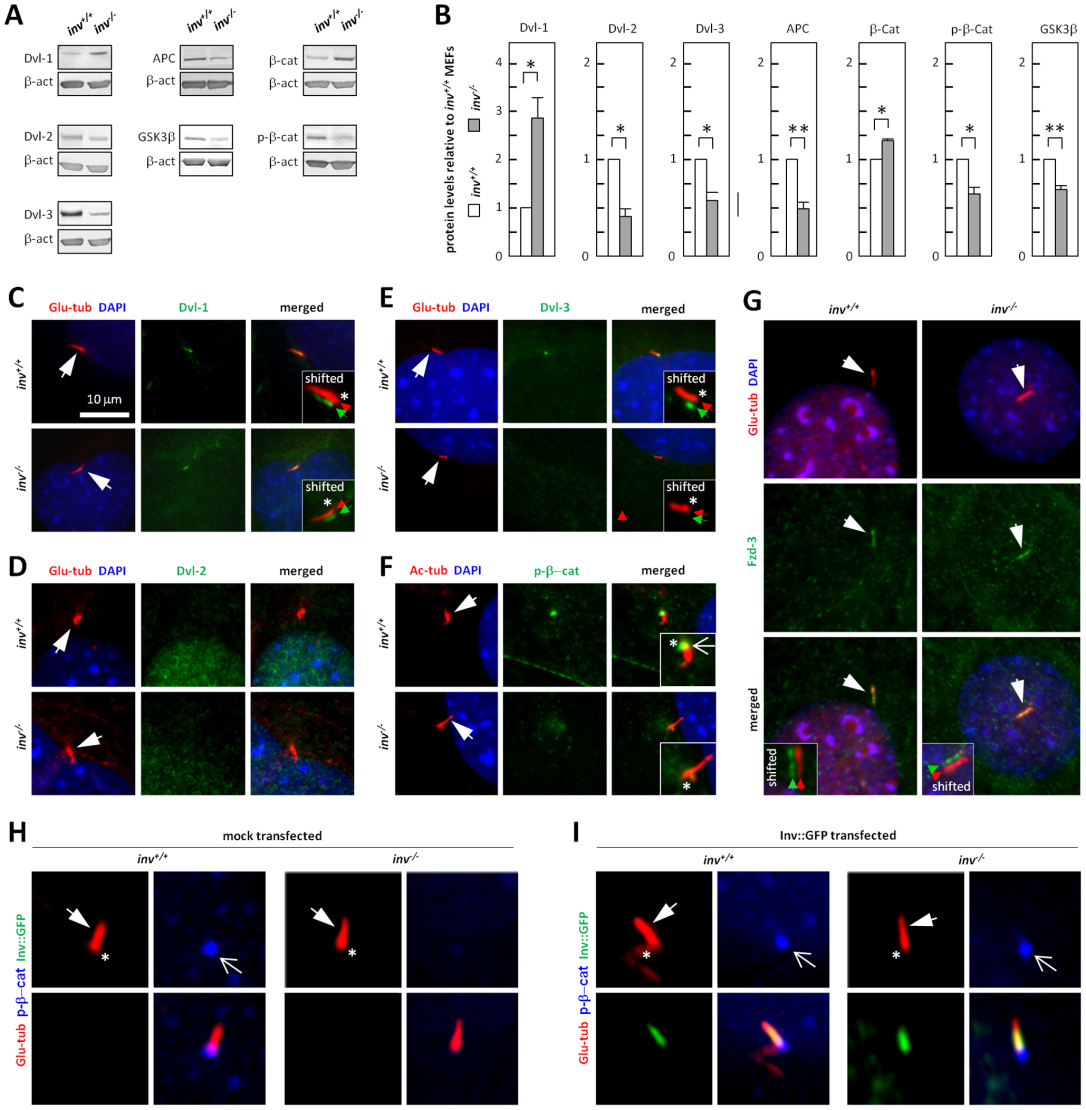
Wnt signaling is dysregulated in *inv^−/−^* MEFs. (**A**) WB analysis of growth arrested *inv^+/+^* and *inv^−/−^* MEFs with antibodies against Dishevelled (Dvl) -1, -2 and -3, Adenomatous polyposis coli (APC), Glycogen synthethase kinase 3 β (GSK3β), β-catenin (β-cat) and S^45^-phosphorylated β-catenin (p-β-cat). (**B**) Quantifications of WB analysis from (A). Histograms represent mean ± S.E.M. (n≥3). (**C,G**) IFM analysis of growth arrested *inv^+/+^* and *inv^−/−^* MEFs. Primary cilia (Ac-tub or Glu-tub, *red*) are marked with closed arrows, nuclei are stained with DAPI (*blue*). *Green* protein localizations are Dvl-1 (C), Dvl-2 (D), Dvl-3 (E), p-β-cat (F) and Frizzled-3 (Fzd-3) (G). (**H,I**) IFM analysis on the localization of S^33/37^-T^41^-phosphorylated β-catenin (p-β-cat) (*blue*, open arrows) to the ciliary base (asterisks) in mock (H) and Inv::GFP (*green*) (I) transfected *inv^+/+^* and *inv^−/−^* MEFs. Primary cilia (Glu-tub, *red*) are marked with closed arrows.

In order to verify whether *inv^−/−^* MEFs exhibit increased canonical Wnt/β-catenin signaling, WB and IFM analyses were carried out to study the expression, localization and phosphorylation of β-catenin and Dvl-1-3, as well as APC and GSK3β. WB analysis showed that *inv^−/−^* MEFs displayed an increased protein level of Dvl-1, whereas protein levels of Dvl-2, Dvl-3, APC and GSK3β were significantly reduced, compared to *inv^+/+^* MEFs (Figure 3A,B). Further, *inv^−/−^* MEFs showed a reduction in the GSK3β-mediated phosphorylation of β-catenin and, accordingly, an increase in the β-catenin protein level (Figure 3A,B). These results support the conclusion that canonical Wnt/β-catenin signaling is upregulated in *inv^−/−^* MEFs.

Given the reported interaction between Inversin and Dvl proteins [35], we next examined the localization of Dvl-1, -2, and -3 in *inv^+/+^* and *inv^−/−^* MEFs. We detected Dvl-1 and Dvl-3, but not Dvl-2, in the primary cilium (Figure 3C–E). Similarly to Inversin, Dvl-1 localized to the lower part of primary cilia of wt and mutant cells (Figure 3C). In contrast, Dvl-3 was present only at the basal region of the cilium and was, notably, absent in the primary cilia of *inv^−/−^* MEFs (Figure 3E). All three Dvl isoforms were also detected in the cytosol and at the plasma membrane (data not shown). In accordance with [72] there was a distinct accumulation of phosphorylated β-catenin (p-β-cat) at the basal region of the cilium in *inv^+/+^* MEFs. p-β-cat staining was reduced and appeared more dispersed at the ciliary base in *inv^−/−^* MEFs (Figure 3F). We further observed localization of Fzd-3 to primary cilia of both *inv^+/+^* and *inv^−/−^* MEFs (Figure 3G), indicating that Wnt signaling may be regulated directly by activation of receptors in the cilium, and that ciliary targeting of Fzd-3 is independent of Inversin. Finally, expression of Inv::GFP in *inv^−/−^* MEFs restored the distinct accumulation of p-β-cat at the basal region of the cilium (Figure 3H,I), confirming a specific role of Inversin in regulating β-catenin stability at the primary cilium. Taken together, these results demonstrate that *inv^−/−^* MEFs exhibit increased canonical Wnt signaling and changes in the localization of Wnt signaling components at the primary cilium.

### Aberrant Activation and Localization of Rho GTPases in *inv^−/−^* MEFs

**Figure 4:**
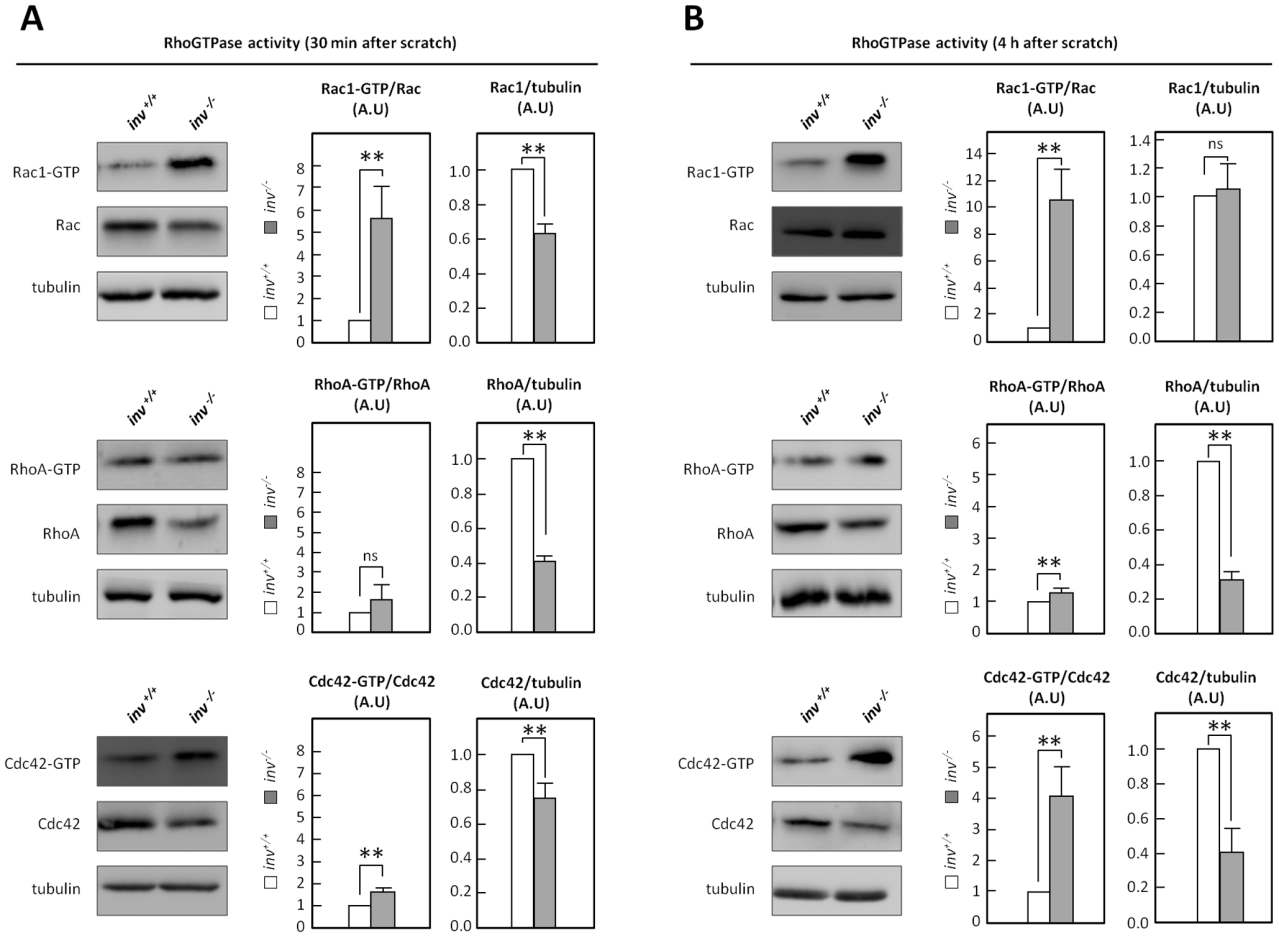
Inversin affects the activity of the Rho GTPases. Activity of Rac-1, RhoA and Cdc42 was determined by pull-down in migrating *inv^+/+^* and *inv^−/−^* MEFs 30 min (A) or 4 h (B) after scratch. Histograms represent the mean ± S.E.M. of three independent experiments.

Rho GTPases are essential regulators of cell motility, in part via the downstream effectors Arp2/3 and WAVE/WASP [43]. To test the hypothesis that Inversin plays a role in regulation of Rho GTPase activity, effector domain-binding assays were employed to estimate the fractions of active Cdc42, Rac1 and RhoA in *inv^+/+^* and *inv^−/−^* MEFs in wound healing assays. 30 min after onset of migration (induced by introducing multiple scratches in the cell culture), a 5-fold increase of Rac1 activity and a small, yet significant, increase in Cdc42 activity were detected in *inv^−/−^* MEFs compared to wt, whereas RhoA activity did not differ significantly between *inv^+/+^* and *inv^−/−^* cells (Figure 4A). Interestingly, upon 4 h of migration, a 4-fold increase in Cdc42 activity and an 11-fold increase in Rac1 activity were observed in *inv^−/−^* MEFs compared to wt, whereas RhoA activity was only slightly, albeit significantly, increased compared to wt (Figure 4B). Notably, the expression levels of Cdc42 and RhoA were markedly decreased in *inv^−/−^* MEFs compared to wt, whereas Rac1 expression was reduced at 30 min yet not at 4 h, after introduction of the wound (Figure 4A–B).

**Figure 5:**
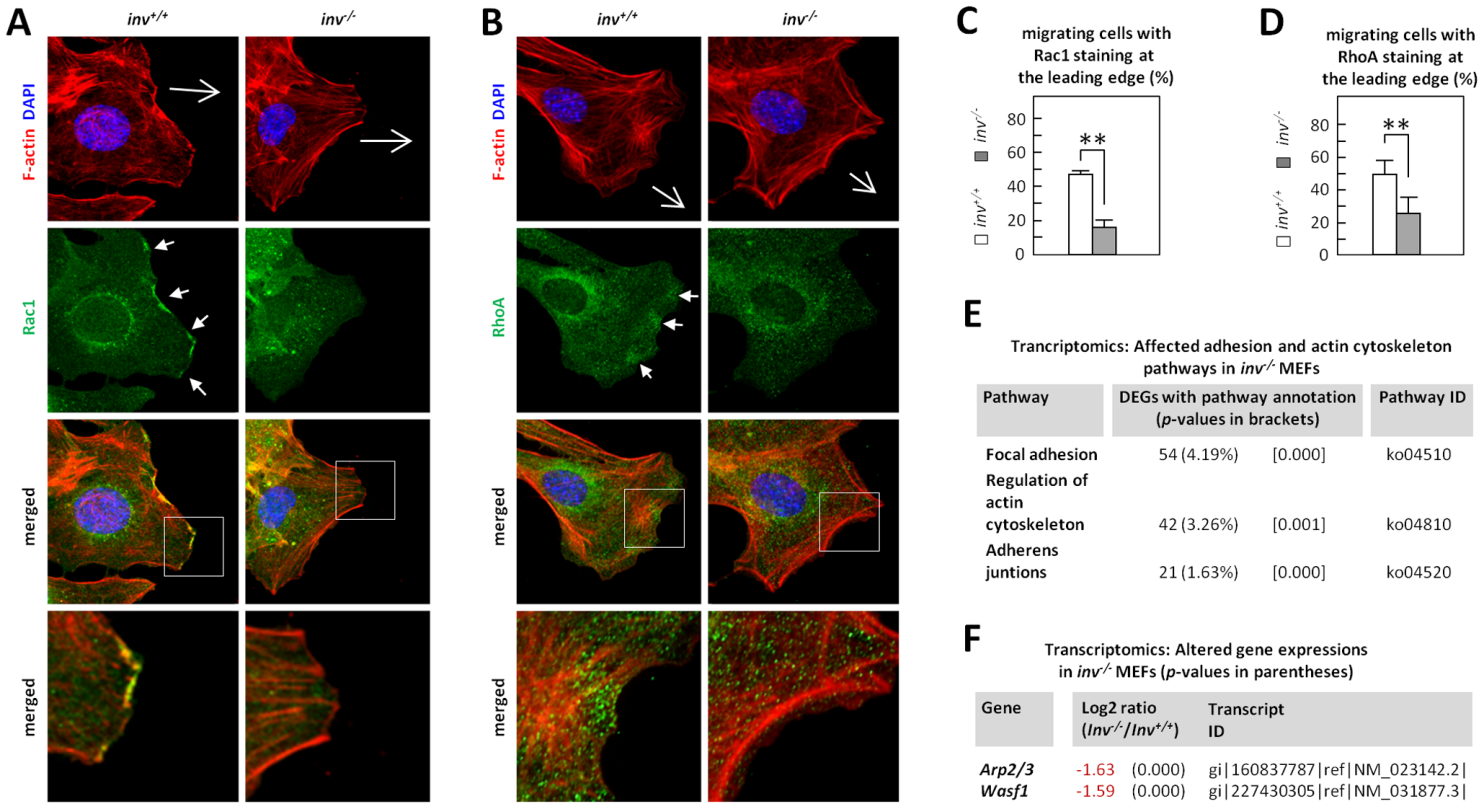
Inversin affects the localization of the Rho GTPases and gene expression in migration-related pathways. (**A,B**) IFM analysis of Rac-1 (A, *green*) or RhoA (B, *green*) localization after 4 h migration of *inv^+/+^* and *inv^−/−^* MEFs. Arrows indicate direction of migration and arrowheads indicate Rac1/RhoA localization at the leading edge. The actin cytoskeleton is stained with phalloidin (F-actin, *red*) and nuclei are stained with DAPI (*blue*). (**C,D**) Quantification of leading edge staining of Rac1 (C) and RhoA (D) in *inv^+/+^* and *inv^−/−^* MEFs represented as mean ± S.E.M. (n = 3). (**E,F**) Transcriptomics of growth arrested MEFs with listed *p* values (n = 3). Migration-related pathways with number and percentage of differentially expressed genes (DEGs) in *inv^−/−^* compared to *inv^+/+^* MEFs (E). Downregulation of specific genes controlling actin polymerization in *inv^−/−^* relative to *inv^+/+^* MEFs (F).

Correct targeting of Rac1 and RhoA at the leading edge is essential for proper directional migration [73]–[75], and we therefore next examined the localization of these proteins by IFM analysis. In *inv^+/+^* MEFs, both GTPases localized diffusely to the cytosol as well as to distinct punctae in cytoplasmic protrusions of migrating cells (Figure 5A,B, left panels). In comparison, *inv^−/−^* MEFs showed a markedly reduced Rac1 and RhoA localization at cell surfaces facing the wound (Figure 5A,B, right panels). Quantitative image analysis confirmed that localization of both Rho GTPases at the leading edge was significantly reduced in *inv^−/−^* MEFs (Figure 5C,D). These results show that while Rho GTPase activity was increased in *inv^−/−^* MEFs, the defective targeting of Rac1 and RhoA in these cells could compromise the organization of the cytoskeleton required for cell migration. Indeed, transcriptome analysis revealed a large extend of deregulated gene expression within pathways regulating focal adhesions, the actin cytoskeleton and adherens junctions in *inv^−/−^* MEFs (Figure 5E). In this regard, expression of *Wasf1* (encoding Wave1) and *Arp2/3*, both of which are regulated by Rac1 and Cdc42 and essential for actin reorganization during lamellipodia formation [43], were significantly downregulated in *inv^−/−^* MEFs (Figure 5F). These findings demonstrate that Inversin regulates the activation and localization of Rho GTPases and the expression of a large number of key proteins in cell migration.

### Expression and Leading Edge Localization of ERM Proteins are Altered in *inv^−/−^* MEFs

**Figure 6:**
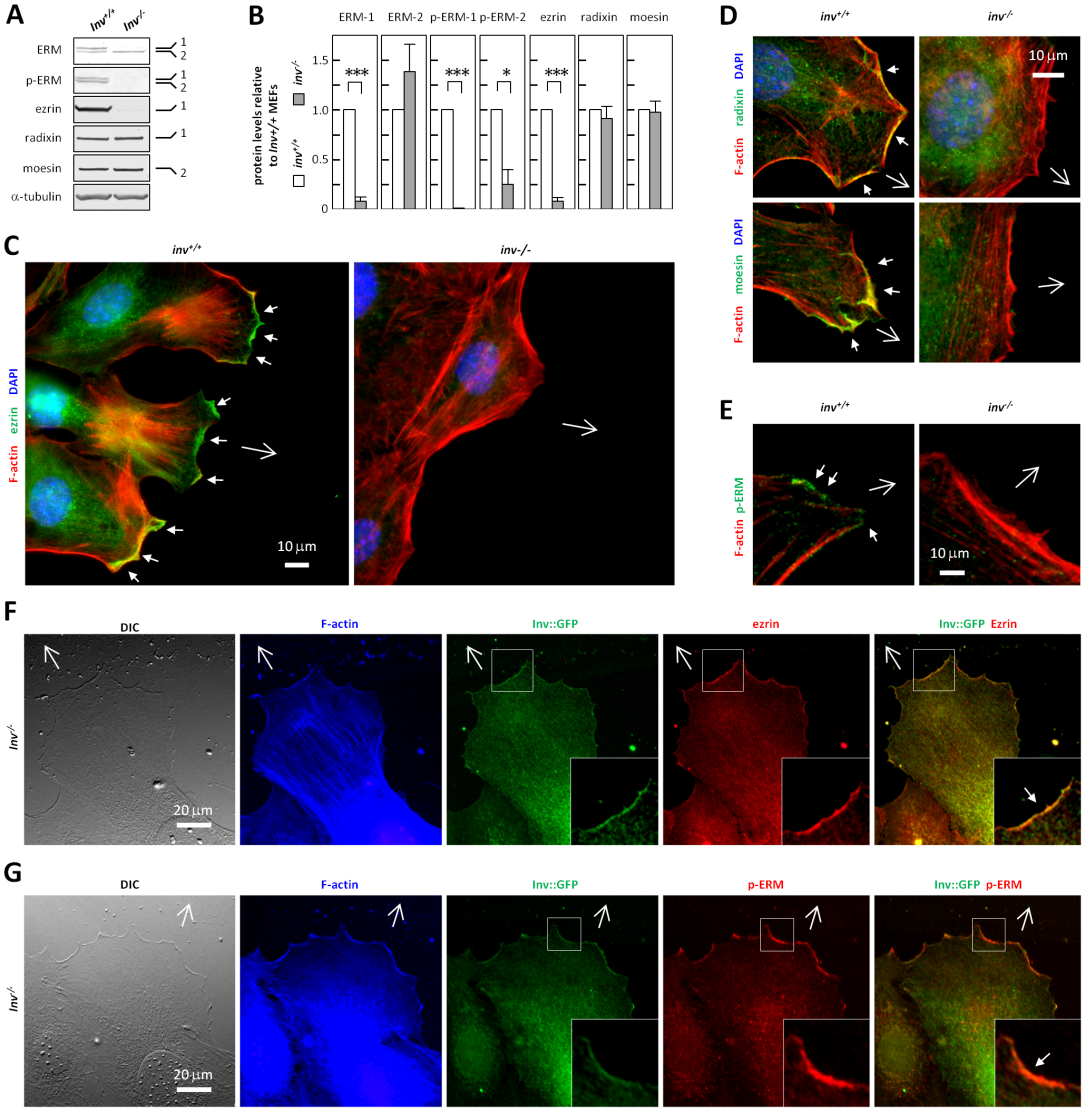
Inversin affects expression, regulation and localization of ERM proteins. (**A**) WB analysis of growth arrested *inv^+/+^* and *inv^−/−^* MEFs with antibodies against total ezrin/radixin/moesin (ERM), phosphorylated ERM (T^567^ of ezrin, T^564^ of Radixin, T^558^ of moesin, p-ERM), ezrin, radixin and moesin, and α-tubulin as control, with indications of the 80 (1) and 75 (2) kDa bands. (**B**) Quantification of WB from (A); Histograms represent mean ± S.E.M. (n≥3). (**C,E**) IFM analysis of growth arrested *inv^+/+^* and *inv^−/−^* MEFs in wound healing assays, with phalloidin staining of the actin cytoskeleton (F-actin, *red*) and nuclei with DAPI (*blue*). Open arrows indicate direction of migration, and arrowhead indicate leading edge staining of ezrin (C, *green*), Radixin (D; upper panel, *green*), moesin (D; lower panel, *green*), and p-ERM (E, *green*). (**F,G**) DIC and IFM analysis on lamellipodium formation and localization of ERM (F, *red*) and p-ERM (G, *red*) to the leading edge of migrating cells in wound healing assays in Inv-GFP (*green*) transfected *inv^−/−^* MEFs. The actin cytoskeleton is stained with phalloidin (F-actin, *blue*). Open arrows indicate direction of migration.

In light of the known roles of RhoA, Rac, and Cdc42 in regulating ERM proteins [44], and the known involvement of ERM proteins in the formation of leading edge lamellipodia [76], we next asked whether *inv^−/−^* MEFs exhibited altered expression and activity of ERM proteins. WB analysis using an antibody recognizing total ezrin, radixin and moesin (ERM) showed an about 10-fold decrease in the proteins level(s) of ezrin and/or radixin (both 80 kDa) in *inv^−/−^* MEFs, whereas the level of moesin (75 kDa) was unaltered. Separate examination of the individual ERM proteins confirmed this observation and showed that the 80 kDa ERM protein with reduced expression in *inv^−/−^* MEFs corresponds to ezrin. Interestingly, ERM protein activation (represented by their phosphorylation at T^567/564/558^) was also markedly reduced in *inv^−/−^* cells (Figure 6B,E).

To investigate whether lack of Inversin also alters ERM trafficking to the leading edge of migrating cells, IFM analysis was carried out in wound healing assays. Typical lamellipodia in the direction of the wound, with prominent cortical F-actin and clear leading edge ezrin localization, were formed in *inv^+/+^* MEFs (Figure 6C, left panel), but in *inv^−/−^* MEFs, ezrin was largely absent at the plasma membrane, in congruence with the low expression of ezrin in these cells (Figure 6C, right panel). Notably, localization of radixin and moesin to wound-approaching cell edges was also absent in *inv^−/−^* MEFs, despite the fact that the mutant cells expressed these proteins at a level comparable to that of wt cells (Figure 6D). Further, phosphorylated ERM proteins were strongly expressed at the leading edge in *inv^+/+^* MEFs, but were absent from the cell edge facing the wound in *inv^−/−^* MEFs (Figure 6E). Finally, expression of Inv::GFP in *inv^−/−^* MEFs restored formation of well-defined lamellipodia in the direction of the wound with localization of both ERM and phospho-ERM to the leading of migrating cells (Figure 6F,G). Collectively, these results support the conclusion that Inversin plays a major role in regulation of the expression, regulation, and leading edge localization of ERM proteins.

### Expression and Leading Edge Localization of NHE1 are Altered in *inv^−/−^* MEFs

**Figure 7:**
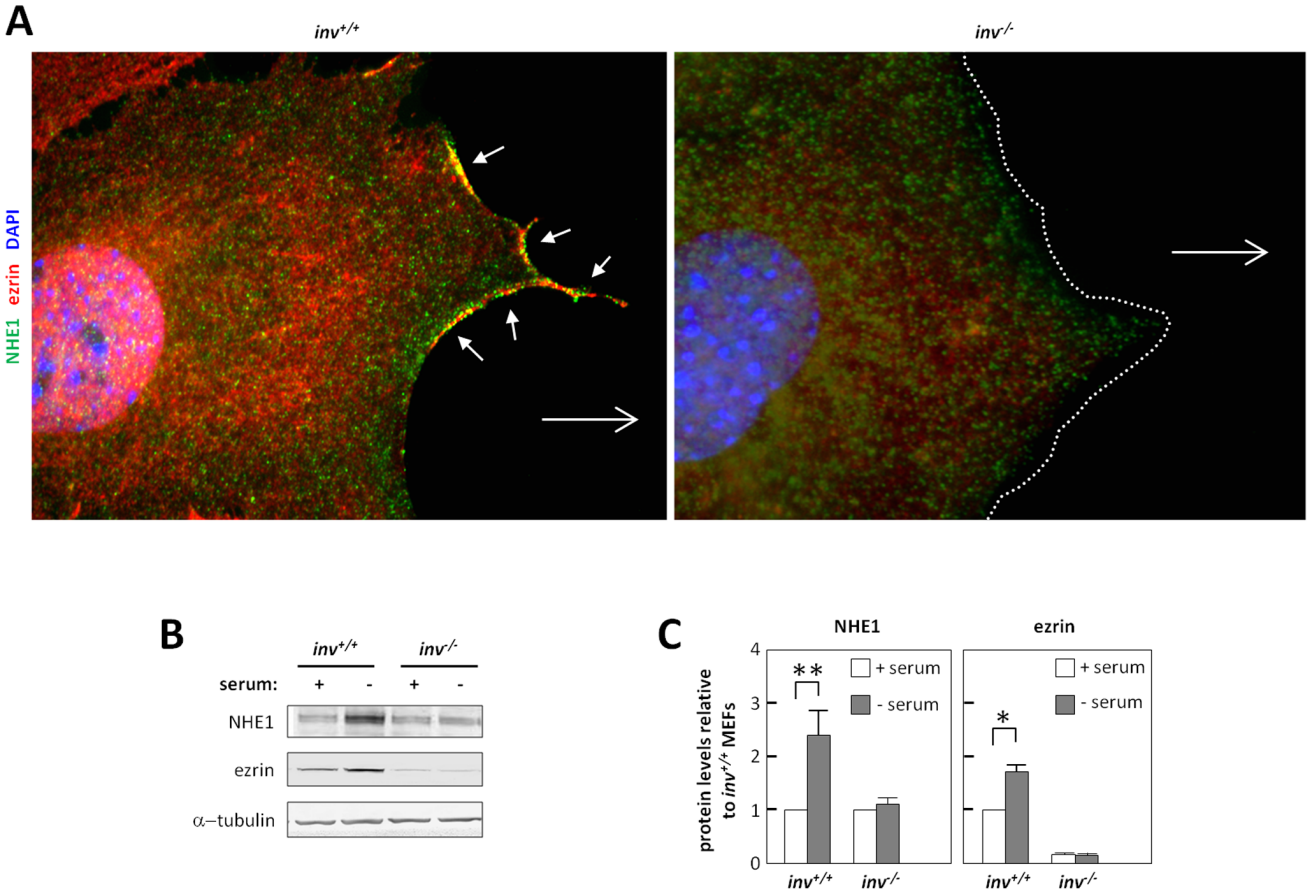
Inversin affects the localization and expression of NHE1 and ezrin. (**A**) IFM analysis of growth arrested *inv^+/+^* and *inv^−/−^* MEFs in wound healing assays. Open arrows indicate direction of movement and arrow heads show leading edge co-localization of the Na^+^/H^+^ Exchanger 1 (NHE1, *green*) and ezrin (*red*). Nuclei are stained with DAPI (*blue*). (**B**) WB analysis of *inv^+/+^* and *inv^−/−^* MEFs grown in the presence (+) or absence (−) of serum, with antibodies against NHE1, ezrin, and α-tubulin as control. (**C**) Quantification of WB from (B) represented as mean ± S.E.M. (n≥3). White dotted line marks the front of *inv^−/−^* MEFs facing the wound.

ERM proteins physically link the actin cytoskeleton to the Na^+^/H^+^ Exchanger NHE1, which we and others have shown to localize to the leading edge and play an essential role in directional cell migration [49], [77], [78] in a partially primary cilium-dependent manner [49], [79]. We therefore hypothesized that NHE1 localization would be disrupted in the *inv^−/−^* cells. IFM analysis of quiescent cells in wound healing assays confirmed targeting and colocalization of NHE1 with ezrin at the leading edge of wt MEFs, (Figure 7A, upper panel). In contrast, NHE1 was absent from cell edges facing the wound of *inv^−/−^* MEFs (Figure 7A, lower panel). We also performed IFM analysis on cells cultured at low confluence, allowing them to move freely with no contact to neighboring cells. In these experiments, *inv^+/+^* MEFs formed lamellipodia with clear leading edge localization of NHE1, which colocalized with ezrin (Figure S2A,B, left panels), whereas *inv^−/−^* MEFs showed no detectable localization of either protein at the cell surface (Figure S2A,B, right panels). In accordance with our previous findings [49], NHE1 was upregulated during growth arrest in *inv^+/+^* MEFs, to more than twice the level in cycling cells. Notably, this upregulation was abolished in *inv^−/−^* MEFs (Figure 7B,C). Similarly, we observed a significant, growth arrest-associated upregulation of ezrin in *inv^+/+^* MEFs, but not in *inv^−/−^* MEFs (Figure 7B,C). Collectively, the data in Figure 6 and 7 show that Inversin regulates both the cell-cycle-dependent expression and the lamellipodial targeting of ERM proteins and NHE1, as well the phosphorylation of ERM proteins.

## Discussion

We show here that Inversin plays a critical role in regulation of cellular pathways associated with polarity control in cell migration. *Inv^−/−^* MEFs exhibit marked defects in cell migration, in conjunction with defective orientation of primary cilia towards the leading edge, altered Wnt signaling, and marked changes in motility-related regulation of Rho GTPases, ERM proteins, and NHE1. In addition, Inversin deficiency elicited marked changes in the RNA expression profiles of genes involved in the regulation of cytoskeletal organization, including the NHE-regulatory factor, *Nherf1,* as well as *Arp2/3*, *Wave1*, and clusters of genes regulating formation and function of adherence junctions and focal adhesion. These findings are the first to characterize, at the single-cell level, the roles of Inversin in regulating transcriptional processes and polarity pathways during cell migration.

*Inv^−/−^* MEFs exhibited reduced migration speed and defects in lamellipodia formation in wound healing assays. As previously reported for fibroblasts [45], [46], primary cilia in *inv^+/+^* MEFs were positioned in front of the nucleus, pointing towards the leading edge and in parallel to the path of migration. This was in sharp contrast to *inv^−/−^* MEFs, in which primary cilia were randomly positioned and lacked an axis of orientation towards the edge of the wound. On sensory cells in the vertebrate inner ear, PCP signaling controls the position of the kinocilium, which then directs the organization of the stereocilia [34], [80]. In line with previous findings [45], [49], we suggest that ciliary orientation and positioning are critically linked to the polarity of migrating cells and that Inversin plays a significant role in regulating these processes.

Since Inversin acts as a molecular switch between the β-catenin and PCP pathways [35], [36], we hypothesized that ciliary orientation might be regulated by Inversin through the facilitation of non-canonical Wnt/PCP signaling. In order to investigate this in further detail, we carried out gene and protein expression analysis on Wnt signaling components in *inv^+/+^* and *inv^−/−^* MEFs. Our transcriptome analysis revealed a dramatic change in expression of several genes in both canonical and non-canonical Wnt signaling. Most importantly, there was a major up-regulation of multiple β-catenin target genes, confirming that Inversin deficiency leads to up-regulation of canonical Wnt signaling. As an example, *Gsc*, a canonical Wnt-responsive gene encoding Goosecoid [81] that controls cell migration in *Xenopus* embryos [82], was upregulated more than 1,500-fold in *inv^−/−^* compared to *inv^+/+^* MEFs. In concurrence with these data, our protein expression analysis confirmed up-regulation of canonical Wnt signaling in *inv^−/−^* MEFs as judged by increased protein levels of Dvl-1 and β-catenin, consistent with a reduction in S^33/37^-T^41^-phosphorylation of β-catenin at the primary cilium. Further, the protein levels of Dvl-2 and Dvl-3 were significantly reduced in *inv^−/−^* MEFs, and this was associated with a prominent reduction in ciliary accumulation of Dvl-3 at the ciliary base. These results suggest that Inversin partly acts through Dvl-3 at the cilium to control PCP signaling. Indeed, Dvl proteins critically regulate the positioning of the centrosome during cell migration [83] and it was recently shown that defects in accumulation of Dvl-3 at the ciliary base leads to loss of PCP [84]. Hence, the aberrant positioning and orientation of the primary cilium observed in *inv^−/−^* MEFs may relate to reduced expression and lack of localization of Dvl-3 to the primary cilium in these cells.

In terms of Dvl protein function, Schlessinger and colleagues identified a link between the Cdc42/Par6/aPKC and Wnt5a/Dvl-2 pathways in the form of an increased interaction between aPKC and Dvl-2 during wounding of cell cultures, suggesting cooperation between these two pathways to induce cell polarization and migration [83]. Further, Cdc42, which exhibited increased activity yet strongly decreased localization at cell surfaces facing the wound in *inv^−/−^* MEFs compared to *inv^+/+^* MEFs, is an essential regulator of directional cell motility and is implicated, with the Par6/aPKC complex, in reorientation of the centrosome to face the leading edge [74], [85], [86]. In accordance with our findings, increased activation of Cdc42 in MEFs attenuated centrosome reorientation toward the leading edge and migration during wound healing [87]. It can thus be speculated that in *inv^−/−^* MEFs, the reduced Dvl-2 level could lead to insufficient aPKC/Dvl-2 interaction. In response, the Cdc42/Par6/aPKC signal transduction may compensate, leading to an over-activation of Cdc42, which in turn may induce the inhibition of centrosome reorientation and migration observed in *inv^−/−^* cells.

Wnt/Fzd/Dvl signalling can directly stimulate the activity of Rac, Cdc42 and RhoA [38], [88], and Cdc42 can secondarily activate Rac via the recruitment of the Rac-GEF βPIX [74]. Hence, we hypothesize that the Rho GTPase dysregulation in *inv^−/−^* MEFs is at least in part downstream from the observed changes in Wnt signaling. Consistent with the role of Rac in control of cellular directionality and velocity [73], [89], and the reported link between elevated Rac activity and loss of fibroblast motility [90], we detected strongly increased Rac1 activity in *inv^−/−^* MEFs. Interestingly, a similar decrease in Rac1 expression accompanied by an increased activity was observed in primary vascular smooth muscle cells treated with siRNA against Nherf1 [71], which we found downregulated in *inv^−/−^* MEFs. The high level of activation of Cdc42 and Rac1 in the *inv^−/−^* MEFs could in turn suppress RhoA activity [87], [90], which may explain why the activity of this GTPase was only slightly increased in *inv^−/−^* cells. Despite the modest effect on RhoA activity, *inv^−/−^* MEFs exhibited extensive formation of actin bundles and altered focal adhesion patterns compared to wt cells. The dramatic loss of *Wasf1* and *Arp2/3* RNA in *inv^−/−^* MEFs may result directly from the absence of Inversin and dysregulated β-catenin signaling, or may be a negative feedback effect of the elevated Rac1 activity in these cells. Whatever the precise mechanism, it seems likely that the loss of these two essential regulators of lamellipodium formation plays a major role in the impaired formation of lamellipodia in *inv^−/−^* MEFs.

Rho GTPases and ERM proteins reciprocally regulate each other [44], [91], hence, the defective ERM protein activation in *inv^−/−^* MEFs could contribute to the abnormal Rho GTPase activity; a relation which would be novel in the context of Inversin-Wnt signaling. ERM proteins, in turn, play essential roles in regulation of cell polarity and motility, and directly link the actin cytoskeleton to NHE1. We and others have shown NHE1 to play essential roles in directional fibroblast motility, at least in part through its effects on intra- and extracellular pH in the leading edge region [92]–[94] and through signaling in the primary cilium [49], [50]. In this regard, PDGFRαα signaling in the cilium was shown to regulate targeting of NHE1 to the leading edge partly through the AKT pathway that initiates NHE1 translocation to the edge and partly through the MEK1/2-ERK1/2-p90^RSK^ pathway that controls the spatial organization of NHE1 translocation and incorporation and therefore specifies the direction in which the leading edge forms [50]. Indeed, inhibition of the AKT pathway leads to defective cell migration [50] similarly to what is observed for *inv^−/−^* MEFs, suggesting a potential cross-talk between ciliary PDGFRαα and Inversin-mediated Wnt signaling in coordination of NHE1 translocation and directional cell migration. Further, the parallel dysregulation of NHE1 and ezrin expression and localization in *inv^−/−^* MEFs, in conjunction with their known physical interaction and the strong migration defective phenotype observed in these cells, are in accordance with the existence of a tight functional link between NHE1 and ezrin that is important in regulation of cell motility. Studies in *Drosophila melanogaster* have shown that dysregulation of Na^+^/H^+^ exchange activity leads to PCP defects due to defective recruitment of Dvl to Fzd in the plasma membrane [95]. In this context, it is noteworthy that the absence of Inversin is accompanied by a decreased expression of *Nherf1*, which binds F-actin to regulate ezrin and RhoA activity [70], [71] and to suppress Wnt/β-catenin signaling [68]. Furthermore, Nherf1 was reported to localize to pseudopodial tips along with NHE1 and stimulate NHE1 activity, leading to increased invasiveness of breast cancer cells [96], and loss of Nherf1 enhances Wnt/β-catenin signaling, which is associated with hyperproliferation in breast cancer [68].

In conclusion, loss of Inversin abolishes directional migration of MEFs in a manner correlated with dysregulation of Wnt signaling, Rho GTPases and ezrin activity, F-actin organization and with loss of localization of Rho GTPases, ERM proteins and the Na^+^/H^+^ exchanger NHE1 to the leading edge. In conjunction with the known roles of these proteins in regulation of cell motility, our findings suggest the existence of a signaling axis consisting of Inversin, Wnt, Rho GTPases, ERM proteins, and NHE1 that may contribute importantly to the regulation of fibroblast polarization and motility, in a manner partly associated with the primary cilium.

## Supporting Information

Figure S1 **qPCR analysis of** ***Inversin (Invs)*** **mRNA expression in** ***inv^−/−^*** **relative to** ***inv^+/+^*** **MEFs.** (A) Data are presented as mean ± S.E.M. (n = 3).
(TIF)

Figure S2 **DIC and IFM analysis of low-confluent, serum starved** ***inv^+/+^*** **and** ***inv^−/−^*** **MEFs. (A,B)** The actin cytoskeleton is stained with phalloidin (F-actin, blue), and arrowheads indicate cortical localization of NHE1 (A, B, *green*) and ezrin (B, *red*). In (A), microtubules are detected with anti-α-tubulin (α-tub, *red*).
(TIF)
