# Supplementary figures and images for: Inversin/Nephrocystin-2 Is Required for Fibroblast Polarity and Directional Cell Migration
Source: PLoS One. 2013 Apr 8;8(4):e60193. doi: 10.1371/journal.pone.0060193 (PMC3620528; doi:10.1371/journal.pone.0060193)

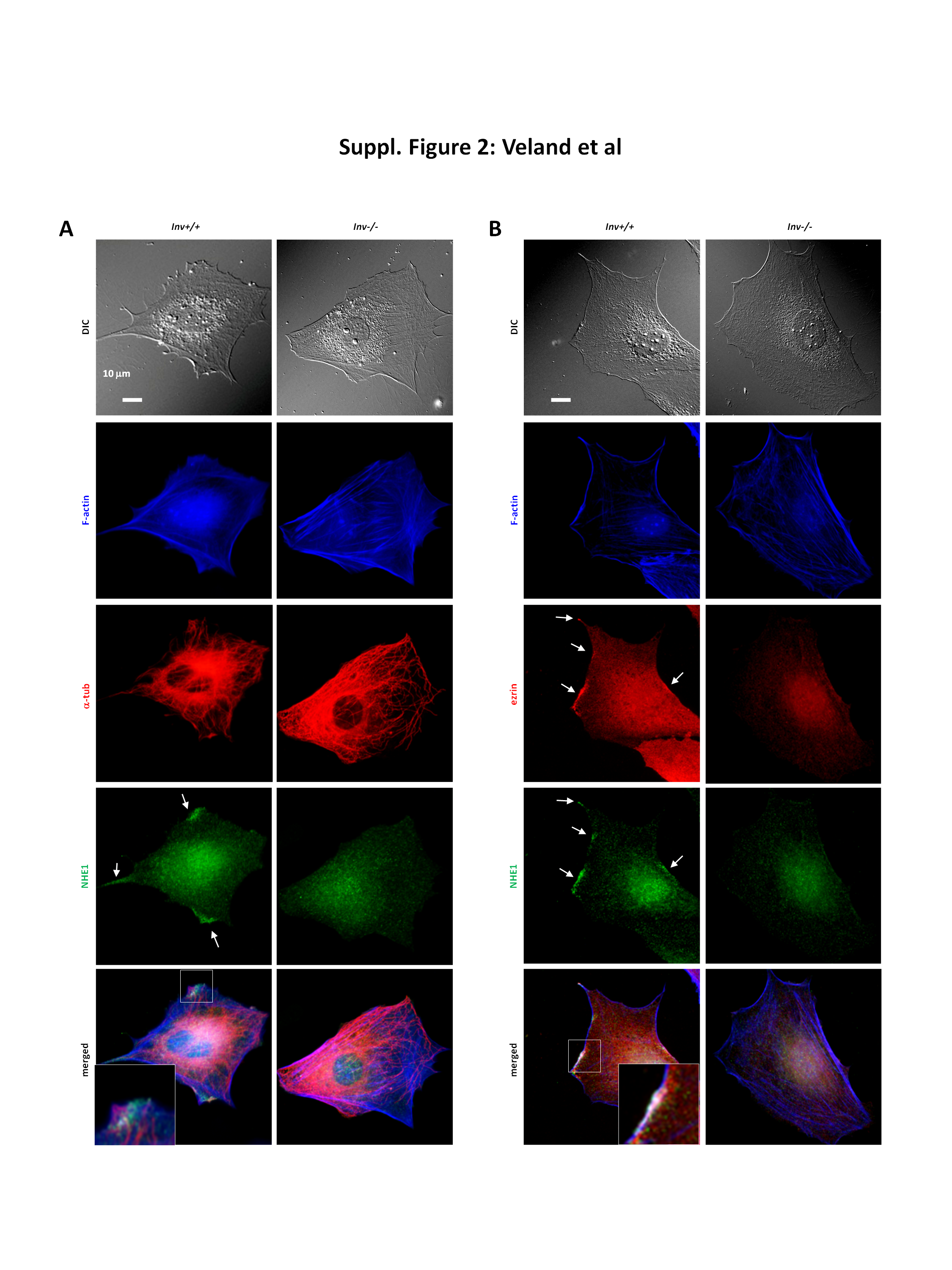

Supplement: Figure S2 — DIC and IFM analysis of low-confluent, serum starved inv+/+ and inv−/− MEFs. (A,B) The actin cytoskeleton is stained with phalloidin (F-actin, blue), and arrowheads indicate cortical localization of NHE1 (A, B, green) and ezrin (B, red). In (A), microtubules are detected with anti-α-tubulin (α-tub, red). (TIF) [file pone.0060193.s002.tif]
